# Examining the association between digital technology literacy and technology-based teaching design: the mediating effect of technology trust and technological self-efficacy

**DOI:** 10.3389/fpsyg.2026.1858642

**Published:** 2026-05-25

**Authors:** Meng Zhang, Lei Liu, Xiaoquan Pan

**Affiliations:** 1 College of Education, Zhejiang Normal University, Jinhua, China; 2 Xingzhi College, Zhejiang Normal University, Jinhua, China; 3 College of Foreign Languages and International Education, Shanghai Jian Qiao University, Shanghai, China

**Keywords:** digital technology literacy, mediation, primary school English teacher, technological self-efficacy, technology trust, technology-based teaching design

## Abstract

As technology becomes increasingly ubiquitous in education, digital literacy has emerged as a prerequisite for educators aiming to enhance teaching practices through technological integration. However, the psychological mechanisms underpinning the application of digital literacy to pedagogical design require further investigation. This study examined the association between digital literacy and technology-based teaching design among a sample of 408 primary school English teachers in eastern China, conceptualizing technology trust and technological self-efficacy as parallel and sequential mediating variables. Structural equation modeling analyses revealed that digital literacy shows a significant positive association with teaching design. This association manifests indirectly through two pathways: the direct path through technology trust and the sequential path through technology trust enhancing technological self-efficacy, which further impacts design. Furthermore, the finding that technology trust is significantly associated with technological self-efficacy underscores their interconnectedness in translating competence into practice. These findings elucidate the ‘literacy→trust→efficacy→practice’ transformation pathway, offering a more nuanced psychological account of digital literacy translation within established frameworks, and providing empirical evidence to inform the development of targeted teacher professional development programs and support platforms.

## 1 Introduction

Driven by next-generation digital technologies (e.g., 5G, AI, big data), digital literacy has become a core competency for educators, crucially enhancing instructional design, pedagogy, and adaptive learning environments (Hu et al., 2003; Teo, 2010; Ifenthaler et al., 2024). While effective educational digital transformation depends critically on teacher acceptance beyond top-down mandates (Davis, 1989; Smarkola, 2007), existing research, grounded in frameworks like TAM, Social Cognitive Theory, and Diffusion of Innovations, often lacks generalizability to authentic settings—particularly resource-constrained contexts—and has not fully articulated the mediating roles of technology trust (TT) as an affective assurance distinct from cognitive beliefs, and technological self-efficacy in teachers’ integration pathways (Teo, 2009; Robertson, 2007).

Addressing these gaps, this study investigates how teachers’ digital literacy relates to instructional technology integration, specifically examining the mediating roles of technology trust (conceptualized as a belief in the reliability, safety, and effectiveness of technology tools, distinct from their perceived usefulness or ease of use embodied in TAM’s core components) and technological self-efficacy (perceived capability to effectively use technological tools). In contrast to TAM’s primary focus on cognitive antecedents of acceptance (PU, PEOU), the model of this study integrates the mediating roles of both affective and efficacy-belief factors, proposing a sequential associative pathway of ‘literacy → trust → efficacy → practice’ mechanism. This integration highlights the critical function of trust in transforming literacy into confident action and efficacy. Theoretically building upon and extending Social Cognitive Theory and the conceptual foundations of Technology Trust, the research highlights how positive tech experiences reinforce self-efficacy (Bandura, 1986). Thus, the primary contribution of this study lies in unpacking the distinct and interconnected psychological mediators that bridge DTL and TBTD in an educational setting by specifically highlighting TT’s role and its influence on TSE. Findings offer practical guidance for teacher development (e.g., structured training to build trust) and tool design. The study advocates a policy shift from hardware provision toward fostering a cultural ecology enabling effective technology appropriation (Venkatesh et al., 2003), providing solutions to counter “idle equipment” and “superficial technology” issues, thereby elucidating the internal mechanisms translating digital literacy into practice.

## 2 Literature review and hypothesis development

The rapid advancement of digital technologies, particularly mobile internet, artificial intelligence (AI), and big data, is transforming education (Bose et al., 2025; Ciarli et al., 2021). This fosters novel pedagogical models like blended learning and personalized instruction (Bates, 2019; Siemens and Long, 2011). Thereby, Digital Technology Literacy (DTL) has become essential for effective engagement and innovation. Conceptually, DTL and TSE occupy distinct positions in the technology integration pathway. DTL pertains to perceived digital technology literacy acquired through training and experience, such as knowledge of tools, analytical skills for information processing, and technical fluencies (Falloon, 2020; Stolpe and Hallström, 2024). In contrast, TSE reflects subjective confidence in one’s ability to deploy technology effectively in dynamic pedagogical contexts (Bandura, 1997), influenced by mastery experiences and psychosocial supports. While DTL enables task execution, TSE drives behavioral intention and persistence amid challenges (Ertmer et al., 2012). Empirical evidence confirms their discriminant validity (Aesaert and Van Braak, 2015), supporting their treatment as related but non-redundant constructs. Constructivist theory highlights technology’s role in creating authentic problem-solving scenarios (Jonassen, 2000), where higher DTL facilitates mastery of tools and information extraction, strengthening knowledge construction (Li and Tsai, 2014). Connectivism views learning as connecting information nodes, with technology as a connective thread (Downes, 2007). DTL enables efficient navigation and connection-building within knowledge networks (Siemens, 2005), and educators with high DTL better guide students in this process (Scherer et al., 2019).

Educators’ DTL directly impacts technology-enhanced instructional quality. Effective integration of technological, pedagogical, and content knowledge (TPACK) is crucial (Yang and Yang, 2026), with DTL underpinning technological knowledge (TK). High-DTL educators excel at designing innovative learning activities (Harris et al., 2009). DTL thus bridges technology and pedagogy, enabling innovative methodologies. Research confirms DTL empowers learners’ autonomous exploration and equips educators to design and implement technology-integrated teaching, driving the shift towards digital paradigms.

Established theoretical models provide further insights. The Technology Acceptance Model (TAM) identifies perceived ease of use (PEOU) and usefulness (PU), linked to technological literacy, as core cognitive determinants of adoption from a predominantly utilitarian perspective (Davis, 1989). Enhanced DTL clarifies technological capabilities and value, reducing uncertainty and potentially fostering a positive assessment (PU/PEOU), which could build technology trust (TT). However, TT is conceptualized here beyond utilitarian evaluations; it encompasses a broader affective and normative belief in the reliability, safety, and security of the technology itself and its providers/operators (Lankton et al., 2015; Flavian et al., 2006). High TT alleviates anxiety, enabling focus on aligning technology with obejectives. While trust can stem from positive assessments of reliability, it reflects a more fundamental sense of security and dependability independent of (though informed by) specific task performance expectation. Online learning studies support this; students’ DTL positively correlates with platform trust (Hsu and Chiu, 2004), as proficiency facilitates interaction and successful experiences, which reinforce both perceptions of usefulness/ease and overall reliability beliefs. Similarly, educators with higher DTL better evaluate technology suitability and are thus more likely to develop trust.

*Hypothesis 1 (H1): Digital Technology Literacy (DTL) is positively associated with Technology Trust (TT).*

A significant relationship exists between DTL and Technological Self-Efficacy (TSE), defined as one’s belief in successfully using technology. Grounded in social cognitive theory, TSE develops from evaluations of abilities shaped by mastery experiences (Bandura, 1997). Extensive evidence shows DTL positively influences TSE across educational populations.

Learner DTL dimensions (e.g., information retrieval, evaluation, creativity) correlate positively with learning-related TSE (Aesaert and Van Braak, 2015), as competency facilitates task success and reinforces capability beliefs. Similarly, educator DTL, particularly tool operation and integration literacy, is associated with teaching-related TSE (Siyam et al., 2025), and mastery experiences build confidence in technology-integrated teaching.

This relationship is reciprocal; fostering TSE promotes DTL development. Teachers with high TSE engage more actively in edtech professional development, enhancing DTL which further strengthens TSE and pedagogical innovation (Hatlevik et al., 2015). TSE also mediates: High educator DTL increases success likelihood with emerging technologies (e.g., AI-assisted systems). Success experiences enhance confidence (TSE), motivating adoption of innovative approaches (Mekheimer, 2025).

*Hypothesis 2 (H2): Digital Technology Literacy (DTL) is positively associated with Technological Self-Efficacy (TSE).*

Within digital education, DTL is essential for educators and learners and profoundly connected to Technology-Based Teaching Design (TBTD), which critically impacts educational effectiveness. High-DTL educators adeptly leverage tools like collaboration platforms and virtual labs to design student-centered, problem-based tasks fostering active knowledge construction (Jonassen, 1999). Conversely, adhering to cognitive load theory (Sweller, 1994), strong DTL enables precise selection and integration of digital resources to optimize presentation and mitigate overload.

DTL is central to contemporary TBTD. Educators use digital resources to enrich content (Celik, 2023) and proficiency empowers exploration of innovative methodologies (e.g., flipped classrooms, gamification) and workflow optimization (Bergmann and Sams, 2012).

AI and big data integration present opportunities and challenges for TBTD, making DTL decisive. Big data analytics enable precise understanding of students, supporting personalized design, but insufficient DTL impedes mastery of software and deriving actionable insights (Chu et al., 2023). Similarly, in AI-assisted teaching, DTL is fundamental for operating systems and designing integrated activities (e.g., personalized guidance via tutors, automated assessment) (Russell et al., 2019).

*Hypothesis 3 (H3): Digital Technology Literacy (DTL) is positively associated with Technology-Based Teaching Design (TBTD).*

Technology Trust (TT), the affective and normative belief in technological reliability, safety, security, and the integrity of its providers/operators, correlates significantly with Technological Self-Efficacy (TSE) in educational technology. Rooted in social cognitive theory (Bandura, 1997), TSE formation is influenced by environmental factors with a trustworthy technological environment constituting key situational support. Individuals trusting technology perceive stable operation and effective feedback as a secure platform, reducing risk concerns and improving TSE. Learners trusting platforms demonstrate stronger activity-related TSE (Hsu and Chiu, 2004). Literature confirms TT’s positive impact on TSE. For learners, trust in systems indirectly affects efficacy via perceived effectiveness of technological tools (Venkatesh et al., 2003); confident in security and stability, they explore more, strengthening TSE. For educators, TT correlates positively with integration TSE (Ertmer et al., 2012). Trusting devices to be safe and effective motivates training engagement and TSE development. Additionally, provider service quality is crucial; timely support enhances trust, increasing efficacy perceptions and encouraging innovation in technology use (Zhang and Ackerman, 2008).

*Hypothesis 4 (H4): Technology Trust (TT) is positively associated with Technological Self-Efficacy (TSE).*

Technological Self-Efficacy (TSE) critically influences Technology-Based Teaching Design (TBTD). TSE levels correlate with educators’ adoption willingness, implementation intensity, and innovation propensity, acting as a determinant in enacting instructional theories.

Social cognitive theory explains this; self-efficacy governs behavioral choices, leading high-TSE educators to experiment more and identify technology-pedagogy synergies (Bandura, 1997). Constructivist design theory also positions teacher TSE as prerequisite for establishing interactive ecosystems (Jonassen, 1999). Empirically, elevated TSE enables better discernment of congruence between technology affordances and content (Ertmer et al., 2012), aligning with TPACK’s requirement for confidence in knowledge synthesis (Celik, 2023). Advanced technologies demand adaptability, for which TSE provides intrinsic motivation; it develops through experience, predisposing high-efficacy educators to adopt emergent technologies fostering innovation (Mekheimer, 2025; Russell et al., 2019). TSE also indirectly governs design quality motivationally: High TSE educators pursue ambitious goals, while low TSE may avoid complex methods (Pajares, 1992; Albion and Ertmer, 2002). Significantly, successful TBTD implementations enhance TSE, creating positive feedback loops optimizing future designs (Hatlevik et al., 2015).

*Hypothesis 5 (H5): Technological Self-Efficacy (TSE) is positively associated with Technology-Based Teaching Design (TBTD).*

A reciprocal relationship exists between Technology Trust (TT) and Technology-Based Teaching Design (TBTD). The Technology Acceptance Model (TAM) explains that systematic integration occurs when educators trust technology to serve objectives and be operable. Constructivism indicates that achieving technology-supported constructive learning requires trusting technology to convey information and facilitate interaction—a prerequisite for design (Jonassen, 1999). Practically, higher trust positively correlates with adoption willingness (Venkatesh et al., 2003). Trusting educators experiment more readily with combinations, aligning with TPACK’s integration requirement (Celik, 2023). Amid advancing complexity and uncertainty, TT’s importance grows; building trust requires balancing understanding of functions, trust in providers, and risk control (Flavian et al., 2006). High TT alleviates anxiety, enabling focus on aligning technology with objectives.

*Hypothesis 6 (H6): Technology Trust (TT) is positively associated with Technology-Based Teaching Design (TBTD).*

**Figure 1:**
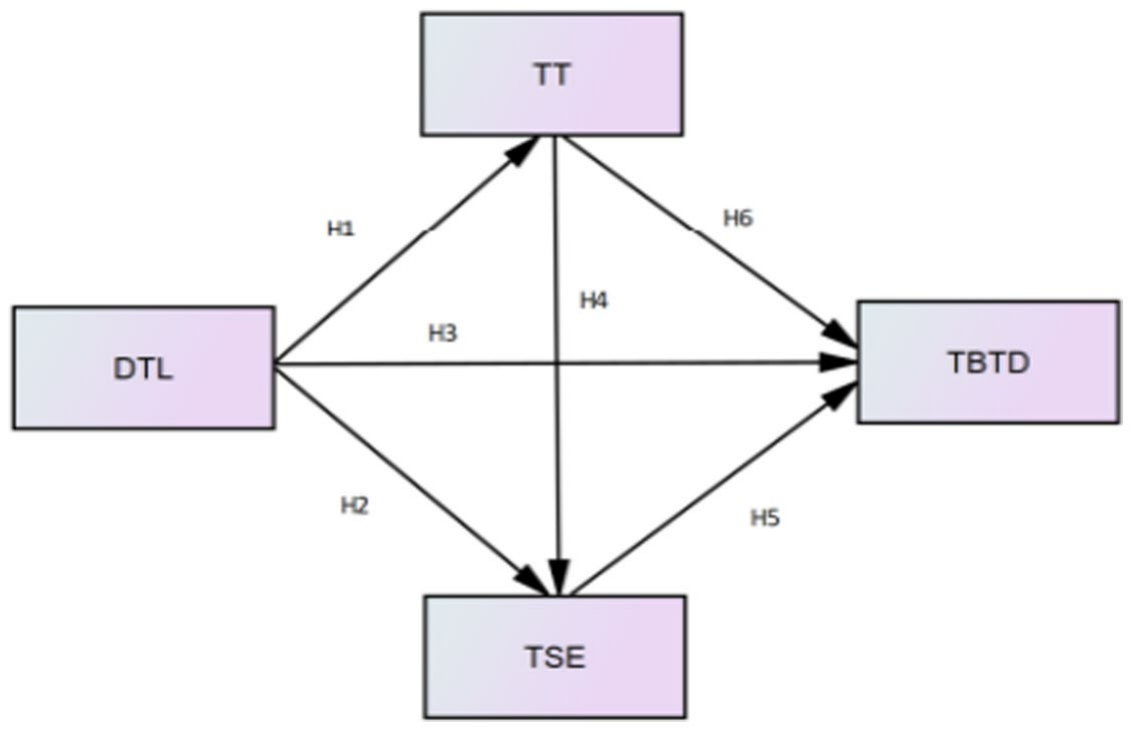
The hypothesized research model.

Synthesizing literature on pedagogical behaviors and factors influencing instructional design in technological environments, this study proposes the hypothesized research model depicted in Figure 1.

## 3 Methodology

### 3.1 Participants and procedures

Using purposive sampling, primary school English teachers from multiple schools in Zhejiang Province in the eastern China, participated in this study. Ethical compliance (informed consent, voluntary engagement, anonymity) was ensured. Data collection via *Wenjuanxing* yielded 431 submissions. After screening, 23 invalid responses with missing data were excluded, resulting in 408 valid questionnaires (94.66% valid response rate). Table 1 details participant demographics.

**Table 1:** Demographic analysis (*N* = 408).

Options	Percentage %	Subtotal
1. Gender		
Male	16.42%	67
Female	83.58%	341
2. Age		
21–25 years old	12.25%	50
26–35 years old	30.64%	125
36–45 years old	41.42%	169
46 years old and above	15.69%	64
3. Level of education		
Postgraduate and above	6.86%	28
Bachelor’s	82.84%	338
Three-year college	10.30%	42
4. Region		
City	71.57%	292
Township	28.43%	116

### 3.2 Measures

Data were collected via a self-report questionnaire assessing four constructs: Digital Technology Literacy (DTL; 9 items), Technology Trust (TT; 3 items), Technological Self-Efficacy (TSE; 5 items), and Technology-Based Teaching Design (TBTD; 5 items), alongside demographics. All items used a 6-point Likert scale (1 = strongly disagree, 6 = strongly agree). Originally developed in Chinese, the questionnaire underwent back-translation following Brislin (1970). Scales were adapted from established literature.

Digital Technology Literacy (DTL) assessed awareness and skills required for effective technology integration (e.g., “I can proficiently use search engine to obtain relative information for instructional design”.). Adapted from Falloon (2020) and Getenet et al. (2024), it comprises awareness (4 items) and skills (5 items) subdimensions. CFA confirmed validity (*χ*^2^/df = 2.839; TLI = 0.935; CFI = 0.956; RMSEA = 0.077; SRMR = 0.057). Cronbach’s *α* is 0.946.

Technology Trust (TT) measured beliefs in the reliability and safety of educational tools (e.g., “I trust that the educational platform adopted by my school is reliable”.). Adapted from Lankton et al. (2015), it focuses on affective/normative assurance distinct from TAM’s utilitarian constructs (Cronbach’s α = 0.847).

Technological Self-Efficacy (TSE) evaluated perceived capability to implement technology-dependent pedagogy (e.g., “I feel confident that I can design a lesson fully reliant on emerging technologies”). Items adapted from Pan (2020) and Bandura (1997) emphasized efficacy in dynamic teaching contexts (α = 0.940).

Technology-Based Teaching Design (TBTD) captured lesson design practices leveraging digital affordances (e.g., “I redesign learning tasks by incorporating interactive digital tools to foster students’ innovative capacity”.). Items adapted from Paquette (2014) and Winkler et al. (2021) emphasized scaffolding, personalization, and problem-solving (*α* = 0.920).

### 3.3 Data analysis

Descriptive statistics were computed using SPSS 26.0. Structural Equation Modeling (SEM) was implemented through AMOS 26.0 in two phases: 1) Confirmatory Factor Analysis (CFA) validated the measurement model, and 2) the SEM structural component estimated relationships between latent variables. Bias-corrected bootstrapping assessed mediated effects (number of bootstrap samples = 5,000). The significance of the mediation effects was assessed using the bias-corrected percentile bootstrap method (Hayes, 2013), computing the confidence interval (CI) for the mediated effect. When zero is not in the CI, it indicates the significance of the indirect effect. To assess the risk of common method bias arising from single-source self-reported data, Harman’s single-factor test (Podsakoff et al., 2003) was conducted to evaluate the extent to which common method bias might inflate observed relationships.

## 4 Results

### 4.1 Descriptive statistics

Table 2 displays descriptive statistics for four latent variables. All means significantly exceeded the scale midpoint (3.5), demonstrating positive attitudes. Standard deviations (0.916–1.079) indicated moderate individual variability. Skewness (−0.723 to −0.400) and kurtosis (−0.313 to 0.348) values conformed to Kline’s (2005) normality thresholds, supporting adequacy for univariate normality assumptions.

**Table 2:** Descriptive statistics of the study constructs.

Constructs	Items	M	SD	Skewness	Kurtosis
DTL	9	4.364	0.945	−0.451	−0.274
TT	3	4.643	0.916	−0.723	0.348
TSE	5	4.467	1.079	−0.607	−0.313
TBTD	5	4.379	0.929	−0.400	−0.145

DTL, Digital Technology Literacy; TT, Technology Trust; TSE, Technological Self-Efficacy; TBTD, Technology-Based Teaching Design.

### 4.2 Test of the measurement model

The measurement model was analyzed using Amos with maximum likelihood estimation (MLE). Reliability and validity were assessed following established criteria (Hair et al., 2011): Cronbach’s *α*, composite reliability (CR), and average variance extracted (AVE) evaluated reliability and convergent validity. Standardized factor loadings (*λ* > 0.7 and significant) and AVE > 0.50 (Teo and van Schaik, 2012) confirmed convergent validity. Table 3 indicates satisfactory reliability and convergent validity.

**Table 3:** Results of the measurement model.

Item	SFL	SE	*t*-value	R^2^	AVE (>0.5)	CR	Cronbach alpha/ McDonald’s ω
Digital technology literacy					0.660	0.946	0.946/0.938
DTL1	0.773	0.054	17.174	0.598			
DTL2	0.848	0.060	19.365	0.718			
DTL3	0.875	0.057	20.214	0.765			
DTL4	0.852	0.058	19.501	0.726			
DTL5	0.803	0.054	18.038	0.645			
DTL6	0.762	0.052	16.851	0.580			
DTL7	0.831	0.057	18.861	0.690			
DTL8	0.776	0.058	17.247	0.602			
DTL9	0.784			0.615			
Technology trust					0.652	0.849	0.847/0.841
TT1	0.821			0.674			
TT2	0.776	0.054	16.571	0.602			
TT3	0.825	0.056	17.704	0.680			
Technological self-efficacy					0.765	0.942	0.940/0.935
TSE1	0.808	0.043	19.690	0.652			
TSE2	0.889	0.044	22.997	0.791			
TSE3	0.929	0.043	24.810	0.864			
TSE4	0.911	0.044	23.971	0.830			
TSE5	0.830			0.688			
Technology-based teaching design					0.700	0.921	0.920/0.917
TBTD1	0.834			0.695			
TBTD2	0.825	0.044	20.125	0.680			
TBTD3	0.880	0.048	22.316	0.775			
TBTD4	0.850	0.050	21.086	0.722			
TBTD5	0.792	0.049	18.931	0.628			

Model fit was evaluated using multiple indices: *χ*^2^/df = 3.855, TLI = 0.922, CFI = 0.933, RMSEA = 0.074, SRMR = 0.063, indicating acceptable fit.

Discriminant validity was assessed by comparing the square root of the AVE (diagonal in Table 4) with the constructs’ inter-correlations. Since the AVE square root for each construct exceeded its highest correlation with any other construct (with correlations ranging up to 0.654), discriminant validity was established. The strong internal consistency (α/*ω* ≥ 0.841) aligned with theoretically expected interrelatedness within constructs, and McDonald’s ω values further confirmed that item responses reflected conceptually coherent yet distinct components of each latent variable.

**Table 4:** Results for the test of discriminant validity.

Constructs	DTL	TT	TSE	TBTD
DTL	(0.812)			
TT	0.592**	(0.807)		
TSE	0.437**	0.454**	(0.875)	
TBTD	0.633**	0.654**	0.574**	(0.837)

**p < 0.01. Diagonal in parentheses: square root of AVE from observed variables (items); off-diagonal: correlations between constructs.

Diagnostic test for common method bias was conducted. Harman’s single-factor test revealed that the first unrotated factor accounted for 35.712% of total variance, which is below the 40% threshold, indicating no common method bias (Podsakoff et al., 2003). Besides, multicollinearity diagnostics were performed. All variance inflation factor (VIF) values ranged from 1.42 to 1.83, well below the conservative threshold of 5 (Hair et al., 2011). Tolerance values (0.55–0.70) exceeded the recommended 0.20 criterion. These results indicate that multicollinearity did not unduly bias parameter estimates in the structural model.

### 4.3 Test of the structural model

As can be seen in Tables 4, a substantial positive correlation has been identified between digital technology literacy and technology trust (*r* = 0.592, *p* < 0.01), thereby providing substantial support for Hypothesis 1 and establishing a robust foundation for the model. Meanwhile, the relevant data provide support for hypotheses 2 and 3 (*r* = 0.437, *p* < 0.01; *r* = 0.633, *p* < 0.01). Significant positive correlations were demonstrated between technology trust and technological self-efficacy (*r* = 0.454, *p* < 0.01) and between technological self-efficacy and technology-based teaching design (*r* = 0.574, *p* < 0.01), thereby providing support for hypotheses 4 and 5. Furthermore, a substantial positive correlation was identified between technology trust and technology-based teaching design (*r* = 0.654, *p* < 0.01), providing additional support for Hypothesis 6.

**Figure 2:**
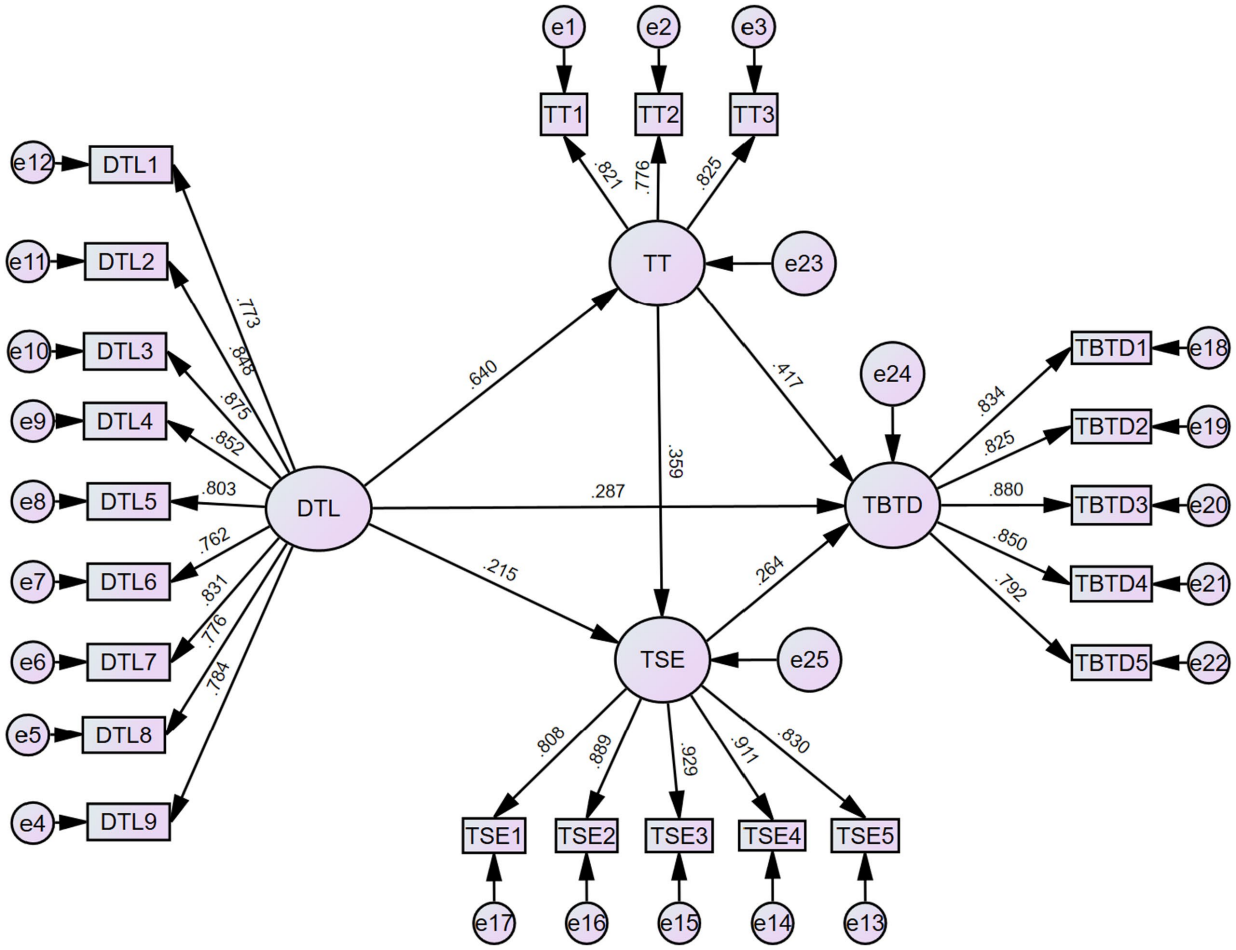
Test results of the final hypothesized model.

Empirical analysis using AMOS (Figure 2; Table 5) supported all six hypotheses (*p* < 0.001). Results suggest that DTL is positively associated with TT (*β* = 0.640, H1) and TSE (*β* = 0.215, H2), establishing its stronger fundamental role in building cognitive trust.

**Table 5:** Hypothesis testing results.

Hypotheses	Path	Path coefficient	*t*-value	Results
H1	DTL-TT	0.640	9.769***	Supported
H2	DTL-TSE	0.215	3.315***	Supported
H3	DTL-TBTD	0.287	5.610***	Supported
H4	TT-TSE	0.359	5.951***	Supported
H5	TSE-TBTD	0.264	6.605***	Supported
H6	TT-TBTD	0.417	8.055***	Supported

***P < 0.001.

Notably, the model underscores the mediating roles of TT and TSE between DTL and technology-based teaching design (TBTD). While DTL is positively associated with TBTD (*β* = 0.287, H3), stronger indirect associations emerge through two paths: DTL increases TT, which both strengthens TSE (*β* = 0.359, H4) and directly enhances TBTD (*β* = 0.417, H6); subsequently, increased TSE also boosts TBTD (*β* = 0.264, H5). TT functions as a key mediator, synergizing with TSE to substantially amplify DTL’s association with teaching design.

These findings reaffirm that TT and TSE act as critical boundary-spanning resources, with DTL requiring conversion into trust and self-efficacy to optimize TBTD, providing quantitative SEM evidence for future research.

### 4.4 Mediational analysis

Structural equation modeling analysis (Table 6) shows that digital technology literacy (DTL) is positively associated with technology-based teaching design (TBTD) via two distinct mediating pathways: through technology trust (TT) (indirect effect = 0.254, 95% CI [0.185, 0.334]) and through technological self-efficacy (TSE) (indirect effect = 0.161, 95% CI [0.111, 0.216]). Besides, the sequential pathway DTL → TT → TSE → TBTD, not displayed in Table 6, is statistically significant based on the sequence pathway test report (*β* = 0.061, *p* < 0.05; 95% CI [0.032, 0.098]), underscoring the theoretically proposed interdependence wherein technology trust correlates with technological self-efficacy, which subsequently facilitates teaching design innovation. Examining these mediating paths independently within the dual-mediation model confirmed their statistical significance and unique contributions. Both indirect effects fall within the conventional range for medium-sized effects (Cohen, 1988).

**Table 6:** Results of the mediational analysis.

From	*β*	Mediator	*β*	To	Indirect effect	95% confidence interval
DTL	0.649	TT	0.417	TBTD	0.254***	[0.185—0.334]
DTL	0.215	TSE	0.264	TBTD	0.161***	[0.111—0.216]

***P < 0.001.

## 5 Discussion

This study examines a sequential-parallel mediation model linking teachers’ digital technology literacy (DTL) to technology-based teaching design (TBTD) through technology trust (TT) and technological self-efficacy (TSE). Results quantify TT and TSE (particularly the TT → TSE link) as critical psychological bridges translating DTL into pedagogical practice, providing a more detailed mechanism within established educational technology adoption and acceptance research, thereby extending technology acceptance frameworks by illuminating the specific role of affective trust and its interplay with self-efficacy and informing educational digital transformation.

TT’s centrality is evident. DTL is positively associated with TT (*β* = 0.640, *p* < 0.001), indicating literacy helps accurate assessment of tools’ reliability and value (Stolpe and Hallström, 2024), thus mitigating perceived risks (Cui et al., 2025). TT’s direct association with TBTD (*β* = 0.417, *p* < 0.001) further confirms its role as a foundation for innovative pedagogical decisions, aligning with TAM/TPACK frameworks (Schmitz et al., 2023). The dominant pathway (DTL → TT → TBTD, *β* = 0.254) underscores that literacy translation hinges on establishing trust. Importantly, the operationalization and findings of TT in the model of this study complement rather than duplicate TAM’s core variables: while PU reflects beliefs about specific task improvement and PEOU relates to effort reduction (Davis, 1989), TT captures a more foundational affective assurance about the technology and system’s dependability and security, regardless of its immediate task-specific benefit (consistent with Lankton et al., 2015). This broader sense of trust lowers perceived risks and provides the psychological foundation for deeper engagement and confidence development, contributing uniquely to our understanding of the psychological pathway from DTL to practice.

TSE functions as an essential complementary mediator and consequence. While DTL is directly associated with TSE (*β* = 0.215, *p* < 0.001), this effect is weaker than DTL → TT, suggesting literacy alone insufficiently builds efficacy. The robust TT → TSE link (*β* = 0.359, *p* < 0.001) empirically supports the SCT proposition that a trusted technological environment provides psychologically safe opportunities crucial for mastery experiences. Furthermore, it highlights how trust translates into confidence. TSE subsequently facilitates overcoming implementation barriers. The sequential pattern DTL → TT → TSE → TBTD suggests interdependence, with fostered trust appearing as a key factor related to efficacy development, which in turn supports the potential for high-quality design outcomes.

The integrated DTL → TT (Assurance) → TSE (Capability Belief) → TBTD (Practice) framework advances application of social cognitive theory and relevant concepts in educational technology contexts, explaining the psychological pathway underpinning integration with greater specificity regarding the distinct mediating roles and their sequence. It clarifies how TT provides the necessary environmental affordance (trustworthy context) enabling the competence embodied by literacy to be transformed into confident practice, resolving part of the “black box” between teacher capability and innovation. By empirically validating this nuanced mechanism, particularly the pivotal role of TT in enhancing both teacher actions (TBTD) and confidence (TSE), this study contributes a finer-grained psychological account to the literature.

Practically, these findings counter “idle equipment” challenges by spotlighting that DTL alone is insufficient. Professional development must therefore cultivate TT via reliable infrastructure, responsive support (Zhang and Ackerman, 2008), and evidence of pedagogical benefits, and strengthen TSE through scaffolded experimentation within such trusted settings. Policymakers should build “trust ecologies” integrating infrastructure, support systems, and innovation recognition to convert literacy and efficacy into effective practice.

Three limitations warrant attention: (1) Cross-sectional design. Exclusive reliance on self-reported cross-sectional data might raise concerns about common method bias, which may inflate correlations among constructs (Podsakoff et al., 2003). Despite diagnostic test indicated no obvious common method bias, findings should be interpreted with awareness that covariation between constructs may be partially attributable to shared method variance. Future research employing multi-trait/multi-method designs or longitudinal behavioral data would provide stronger causal inferences, especially TT-TSE interactions (Kerimbayev et al., 2023). (2) Limited generalizability. Sampling only eastern Chinese primary school English teachers neglects regional/contextual heterogeneity (Robertson, 2007). Replication across diverse populations (regions, subjects, school levels) is needed. (3) Unexamined moderators and uncontrolled variables. School-level support (Hu et al., 2003), teacher autonomy (Smarkola, 2007), and cultural acceptance factors (Venkatesh et al., 2003), as well as uncontrolled demographic variables such as age, education level, region, teaching experience, and professional title, may influence relationships and require investigation.

This study’s findings offer critical guidance for enhancing teachers’ technology integration. First, advancing digital technology literacy is paramount. Second, fostering sustainable integration requires building technology trust via reliable infrastructure, robust technical support, and transparent data security, complemented by showcasing successful pedagogical applications to demonstrate value. Third, technological self-efficacy must be actively developed through scaffolded, hands-on practice (e.g., workshops, peer mentoring) and supported distance learning for under-resourced teachers.

## 6 Conclusion

This study affirms digital literacy’s association with technology-based teaching design, mediated distinctively by technology trust and sequentially through trust-enhanced self-efficacy. Trust emerges as a pivotal catalyst, enabling efficacy development and direct design innovation. These insights refine psychological accounts of technology integration and inform interventions prioritizing trust-building and efficacy support in teacher development. Future studies should test this model’s applicability across diverse educational contexts.

## Data Availability

The original contributions presented in the study are included in the article/supplementary material, further inquiries can be directed to the corresponding author.
